# Metastasis occurring eleven years after diagnosis of human papilloma virus-related oropharyngeal squamous cell carcinoma

**DOI:** 10.3332/ecancer.2014.480

**Published:** 2014-11-13

**Authors:** Jessica Ley, Tanya Wildes, Samir El-Mofty, Douglas Adkins

**Affiliations:** 1 Division of Medical Oncology, Department of Internal Medicine, Washington University School of Medicine, St Louis, MO 63110, USA; 2 Division of Surgical Pathology, Department of Pathology, Washington University School of Medicine, St Louis, MO 63110, USA

**Keywords:** oropharynx, human papilloma virus, p16

## Abstract

Human papilloma virus (HPV)-related oropharyngeal squamous cell carcinoma (OPSCC) is associated with a favourable prognosis, although approximately 20–25% of patients ultimately develop recurrent cancer. Most disease recurrence events appear within 3 years; however, long-term follow-up of reported studies are limited, and the risk of late recurrence is unknown. We present a case report of a patient who developed distant metastases of HPV-related SCC 11 years after initial diagnosis and treatment of HPV-related OPSCC. Late disease recurrence may occur after initial diagnosis of HPV-related OPSCC. This observation has implications on the appropriate duration of follow-up and surveillance of these patients.

## Introduction

Oropharyngeal squamous cell carcinoma of the oropharynx (OPSCC) is an increasingly common subtype of head and neck cancer whose aetiology and prognosis have significantly changed over the last decade [1]. From 1973 to 2004, the overall incidence of OPSCC increased [2]. In 2010, OPSCC was diagnosed in approximately 10,000 patients in the United States [3]. Historically, most cases of OPSCC were due to smoking and alcohol consumption. However, the incidence and prevalence of human papilloma virus (HPV)-related OPSCC has been rising, whereas the incidence of HPV-unrelated OPSCC has been declining [2–3]. The prognosis of patients with HPV-related OPSCC is better than that of HPV-unrelated OPSCC [1, 4–6]. Although most patients with HPV-related OPSCC experience favourable disease-free survivorship with current therapy, approximately 20–25% ultimately develop recurrent cancer and die of their disease [4–6].

The tempo of disease recurrence events appear to differ between HPV-related and HPV-unrelated OPSCC. In HPV-unrelated OPSCC, the majority of disease recurrence events appear within 2 years of completing definitive therapy; whereas, in HPV-related OPSCC, disease recurrence events tend to be delayed in that most appear within 3 years of completing definitive therapy [7]. However, follow-up of large cohorts of patients with HPV-related OPSCC treated on prospective trials and monitored beyond 5 years has not been reported. It is unknown if such patients may be at risk for late disease recurrence. Herein, we describe a patient who developed distant metastases of HPV-related SCC 11 years after initial diagnosis of HPV-related OPSCC.

## Case report

**Figure 1.**
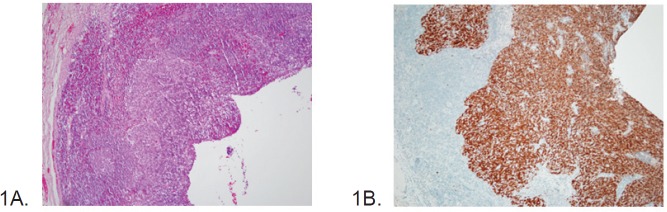
(A) Haematoxylin and eosin (H&E) stain of the neck nodal mass showing nonkeratinized squamous cell carcinoma (×200) in 2000. (B) Strong and diffuse p16 IHC stain of the tumour present in cervical lymph node from 2000.

A 55-year-old male with a limited history of smoking (fewer than ten packs per year) and no history of excessive alcohol consumption presented in March of 2000 with a left level II neck mass as well as a mass at the base of the tongue. Biopsies of the neck nodal mass (Figure 1A) and the base-of-tongue mass revealed non-keratinizing type SCC on haematoxylin and eosin (H&E) stain (x200), a distinct morphologic type that characterizes HPV-related OPSCC [8]. An immunohistochemistry stain (IHC) for p16, a known surrogate marker of HPV [8], was positive (Figure 1B). The patient underwent primary resection of the oropharynx mass and left neck dissection. The pathologic stage of the cancer was T1N2aM0 (IVA). Postoperative adjuvant radiation therapy (7200 cGy) was administered. Surveillance radiologic imaging and clinical examinations continued through 2006, at which time a CT scan of the chest, abdomen, and pelvis did not reveal recurrent cancer.

**Figure 2.**
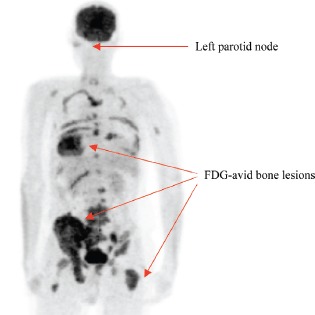
FDG-PET from 2011 showing multiple FDG avid osseous metastatic lesions.

**Figure 3.**
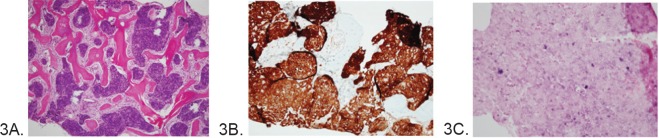
(A) Intraosseous metastasis of nonkeratinizing squamous cell carcinoma in 2011 (H&E ×100), (B) p16 IHC stain of the bone metastasis from 2011, and (C) High-risk HPV ISH of the bone metastasis from 2011 showing positive intranuclear blue stain.

In August 2011, the patient presented with bone pain in the back, pelvis, and legs. Blood work revealed an elevated alkaline phosphatase. Bone and CT scans revealed findings consistent with metastatic osseous lesions, which were confirmed on a fluorodeoxyglucose (FDG)-PET/CT scan (Figure 2). A bone marrow biopsy revealed non-keratinizing SCC on H&E stain (Figure 3A) that was morphologically similar to the OPSCC from 2000 and that was positive for p16 by IHC (Figure 3B) and for HPV by *in situ* hybridization (ISH) for high-risk HPV (Figure 3C). Fibre-optic endoscopy from the nasopharynx to the hypopharynx failed to reveal new oral mucosal lesions. Bronchoscopy and oesophagoscopy were not performed. Anal-rectal examination failed to reveal an anal mass. Palliative chemotherapy was initiated with paclitaxel (135 mg/m2) every 3 weeks and continued for 14 cycles through October 2012 with stable disease. A treatment break was then initiated due to symptomatic peripheral neuropathy. Disease progression documented in February 2013 prompted treatment with Cetuximab.

## Discussion

Long-term follow-up of large cohorts of patients with HPV OPSCC treated on prospective clinical trials and monitored beyond 5 years has not been reported, and thus the risk of late recurrence is unknown [9]. Herein, we report a patient who developed metastatic HPV-related SCC 11 years after initial therapy for HPV-related OPSCC.

To summarize, this patient presented with bone metastases of a p16 positive HPV-related SCC 11 years after the original diagnosis of HPV-related OPSCC. The similarity in the morphology of the pathologic specimens from the primary tumour of 2000 and the metastatic lesion from 2011, the establishment of a HPV relationship by p16 reactivity and/or by HPV ISH in the original primary cancer and in the metastatic bone biopsy specimen, and the failure to find a second cancer by clinical and radiologic exams support that the HPV-related bone metastases originated from the primary HPV-related OPSCC tumour.

A recent report by O’Sullivan *et al* further supports the differences in the tempo of distant relapse events between HPV-related and HPV-unrelated OPSCC. With a median follow-up of 46.8 months, distant relapse events occurred through 5 years following the treatment of HPV-related OPSCC. In comparison, the risk of distant relapse ended at 2 years following treatment in the HPV-unrelated OPSCC group [10].

A review of the literature for other HPV-related cancers, such as cervical and anal SCC revealed that late (>5 years) recurrence events do occur following definitive therapy in these cancers but they are infrequent [11–15]. However, reports of long-term follow-up of large cohorts beyond 5 years following the completion of therapy were also very limited in these other HPV-related cancers.

In patients with a distant history of HPV OPSCC, the development of a subsequent SCC with no obvious primary source should prompt a thorough workup, including oral mucousal and anogenital examination, IHC stain for p16, and ISH for high risk HPV. In the future, genomic profiling of such cases may clearly establish the relationship of the primary HPV OPSCC and the second SCC.

Optimal intervals for systemic radiologic examination to monitor for potential recurrence of OPSCC are not well defined by the review of the evidenced-based literature. A prudent strategy would be to continue to monitor patients with OPSCC by history taking and clinical examination once or twice a year beyond 5 years following completion of treatment. Each encounter would be used to identify signs or symptoms worthy of further evaluation that are concerning for potential recurrence of OPSCC or interim development of second primary cancers. These events would most likely occur in patients who presented with large volume (T4 or N2c or N3) OPSCC or those who have a history of greater than ten pack years of smoking or excessive alcohol consumption.

## Conclusion

This case report illustrates the possibility that HPV-related OPSCC may unexpectedly recur more than 5 years after initial therapy. This observation has important implications regarding the duration of monitoring of patients after completion of therapy. Late (>5 years) recurrence patterns should be prospectively monitored in clinical trials of patients with HPV OPSCC
